# Adverse events from pharmacopuncture treatment in Korea: A protocol for systematic review and meta analysis

**DOI:** 10.1097/MD.0000000000025107

**Published:** 2021-03-19

**Authors:** Jae Eun Park, Sohyeon Kang, Bo-Hyoung Jang, Yong-Cheol Shin, Seong-Gyu Ko

**Affiliations:** a Department of Global Public Health and Korean Medicine Management, Graduate School; b Department of Preventive Medicine, College of Korean Medicine, Kyung Hee University, Seoul, Republic of Korea.

**Keywords:** adverse effects, adverse events, pharmacopuncture, protocol, risk, safety

## Abstract

**Background:**

Pharmacopuncture is a combination of acupuncture and herbal medicine, which involves the injection of herbal extracts into acupuncture points (acupoints). Pharmacopuncture has become one of the major therapeutic tools used in Korea; however, safety is one of the major concerns associated with it. We aim to systematically review clinical studies on the adverse events of pharmacopuncture in Korea.

**Methods:**

To collect data on the incidence and characteristics of adverse events (AEs) and to evaluate pharmacopuncture safety, 2 or more researchers will conduct a comprehensive search of pertinent English and Korean databases using the keywords “pharmacopuncture” and “adverse events.” Regardless of the participants’ conditions or treatment types, we will include clinical studies on the AEs of pharmacopuncture. Studies that were not conducted in Korea, and acupoint injections containing Western medications, vitamins, or autologous serum will be excluded from this study. The severity of AEs will be classified using the common terminology criteria for adverse events, and the causality between pharmacopuncture and AEs will be assessed using the World Health Organization-Uppsala Monitoring Centre (WHO-UMC) causality scale. The quality of identifying and reporting the AEs will be assessed using the McHarm scale. The risk of selection bias will be assessed using the Cochrane risk of bias and the risk of bias for non-randomized studies tools. Studies will be assessed for heterogeneity utilizing Higgins's *I*^2^ statistics, and the risk of publication bias will be assessed and expressed in the form of a contour-enhanced funnel plot.

**Results and Conclusion:**

Comprehensive investigation of all types of clinical studies in Korea will provide clearer evidence of the safety of pharmacopuncture. The results of this study will be useful for traditional medical doctors and patients who use such treatments and interventions.

Systematic Review Registration: Open Science Foundation (osf.io/umhyz).

## 1 Introduction

Pharmacopuncture combines herbal medicine and acupuncture to achieve synergistic effects. It is a traditional medical treatment where herbal extracts are injected into body parts, such as acupoints.^[1,2]^ Pharmacopuncture was first developed in China and was later introduced in Korea in the 1960 s. Since then, various herbal injections have been developed. Since the 2000 s, it has become one of the major therapeutic tools used in Korea and is used to treat various diseases including pain diseases, neurological diseases, and gastrointestinal diseases.^[1–3]^ According to a survey that examined the use of pharmacopuncture, 88.0% of Korean medicine doctors reported using pharmacopuncture during 2017.^[4]^

Owing to its prolific use, the safety issues of pharmacopuncture have been raised. Existing research on pharmacopuncture has mainly focused on examining its effectiveness for treating various diseases. However, the safety of pharmacopuncture has not been thoroughly assessed yet.

We will conduct a systematic review of clinical studies focused on pharmacopuncture in Korea to collect data on the frequency and characteristics of adverse events (AEs) and to evaluate its safety.

## 2 Methods

This systematic review has been registered with OSF (Open Science Foundation registries, https://osf.io) [osf.io/umhyz] and funded through a protocol registry. The protocol will follow the Preferred Reporting Items for Systematic review and Meta-Analysis Protocols guidelines.

### 2.1 Study selection

#### 2.1.1 Types of studies

All clinical study types, including randomized controlled trials (RCTs), non-randomized controlled clinical trials (CCTs), cohort studies, before and after studies, and case reports/case series will be included in this study. Animal studies, review articles, and studies that were not conducted in Korea will be excluded. Survey studies will also be excluded as they do not provide individual patients’ disease information and the specific pharmacopuncture treatment regimen.

#### 2.1.2 Types of participants

Regardless of the participants’ underlying condition, studies in which pharmacopuncture is used as the primary treatment and in which AEs may be associated with pharmacopuncture will be included. There are no restrictions on a subject's underlying disease, disease state, age, sex, or language.

#### 2.1.3 Types of interventions

Regardless of the injection or solution type used, all kinds of pharmacopuncture therapy will be included. We will include studies in which pharmacopuncture is used alone or in combination with other therapies. Acupoint injection using Western medications, vitamins, or autologous serum will be excluded.

#### 2.1.4 Types of outcome measures

In this study, the term AE means “adverse outcomes that occur during or after exposure to drugs or other interventions, and may or may not be caused by the intervention.”^[5]^ To identify the characteristics of AEs associated with pharmacopuncture, we will examine the type and frequency of AEs that occur. In **case studies and in case review series**, we will examine the number of cases, target disease, practitioner type, injection method, solution type used for pharmacopuncture, concomitant treatment, AE symptoms (AE type, diagnosis), and severity and suspected causality of AEs. In **audit studies**, we will examine the injection method, solution type, diagnosis, and the severity and causality of AEs. In **RCTs**, we will examine the target disease injection method and solution type of pharmacopuncture intervention, control treatment method, type, diagnosis, severity, and causality of AEs in both experimental and control groups. The incidence rate of AEs will be evaluated per intervention as follows: number of patient(s) who experienced AEs/total number of patients who received the same kind of intervention ∗ 100 (%). In **CCTs**, we will examine the same items according to RCTs, except the incidence rate. The AEs will be sorted according to the common terminology criteria for adverse events.^[6]^

The types of AEs will be categorized according to patients’ symptoms as systematic reaction, skin problems, and others (nonspecific reactions, not an systematic reaction or skin problems), as classified in previous papers on pharmacopuncture.^[7]^

The severity of AEs will be classified as “mild” when symptoms are present but easily tolerated. The severity of AEs will be classified as “moderate” when the symptoms are uncomfortable enough that they interfere with daily activities. Finally, the severity of AEs will be classified as “severe” when work or daily activities cannot be performed.^[6,8,9]^

Causality between pharmacopuncture and the AEs will be assessed according to the World Health Organization-Uppsala Monitoring Centre (WHO-UMC) Causality Scale.^[10]^ AEs will be rated as “probable” when they occur after pharmacopuncture, disappear after withdrawal, and cannot be explained by other conditions or treatments. AEs will be rated as “possible” when they occur after pharmacopuncture, but no information is provided on their disappearance after treatment is stopped, or whether they could have been caused by other diseases or treatments. AEs will be rated as “unlikely” when a causal relationship between the pharmacopuncture treatment and the AE is impossible. AEs will be rated as “conditional/unclassified” when they occur but more data is required before a conclusion can be made. AEs will be rated as “non-assessable/ unclassifiable” when they cannot be evaluated due to insufficient or contradictory information.^[7,11]^

The quality of detection and reporting of AEs will be reviewed using the McMaster tool for assessing the quality of harms assessment and reporting in study reports (McHarm).^[12,13]^ Additionally, a modified McHarm scale will be used, as shown in Table 1, to further define AEs (Table 1).

**Table 1:** Modified McHarm scale for adverse events of pharmacopuncture.

No	Modified McHarm items
1	Were the pharmacopuncture-related adverse events PRE-DEFINED in the Methods section?
1a	If yes, were any of them prespecified with a priori standardized or precise definitions?
2	Were SERIOUS events^∗^ precisely defined?
3	Were SEVERE events^†^ precisely defined?
4	Were all prespecified pharmacopuncture-related adverse events reported?
5	Was the mode of harms collection specified as ACTIVE^‡^?
6	Was the mode of harms collection specified as PASSIVE^§^?
7	Did the study specify WHO collected the information on pharmacopuncture-related adverse events?
8	Did the study specify the TRAINING or BACKGROUND (e.g., Korean medical doctors) of who ascertained the pharmacopuncture-related adverse events?
9	Did the study specify the TIMING and FREQUENCY of collection of the adverse events?
10	Did the author (s) use STANDARD scale (s) or checklist (s) for adverse event collection?
11	Was the NUMBER of participants that withdrew or were lost to follow-up specified for each study group?
12	Was the TOTAL NUMBER of participants affected by harms specified for studies comparing adverse events across two or more arms?
13	Did the author (s) specify the NUMBER for each TYPE of harmful event for studies comparing adverse events across two or more arms?
14	Did the author (s) specify the NUMBER for each TYPE of adverse event for each study group?

∗ An adverse event results in any of the following outcomes: 1) Death; 2) Life-threatening adverse drug experience; 3) Inpatient hospitalization or prolongation of existing hospitalization (for > 24 hours); 4) Persistent or significant incapacity or substantial disruption of the ability to conduct normal life functions; 5) Congenital anomaly/birth defect; 6) An important medical event that may not result in death but is life-threatening or requires hospitalization may be considered as a serious adverse drug experience when, based upon medical judgment, it may jeopardize the patient or subject and may require medical or surgical intervention to prevent one of the outcomes listed in this definition.

† According to the Common Terminology Criteria for Adverse Events.

‡ Researchers sought to collect information on adverse events.

§ Participants are not specifically asked about or tested for the occurrence of adverse events. Rather, adverse events are identified based on patient reports made on their own initiative.

### 2.2 Data sources

Related studies uploaded by January 2020 will be searched in the following databases:

English databases (n = 3): The Cochrane Central Register of Controlled Trials (CENTRAL), MEDLINE (via PubMed), EMBASE;

Korean databases (n = 5): Oriental Medicine Advanced Searching Integrated System, Korean Studies Information Service System, Research Information Sharing Service, National Digital Science Library, and Korean Medical Database (KMBASE);

Other resources: To avoid missing eligible articles, we will manually search Google Scholar and bibliographic references in relevant publications, including The Acupuncture, the Journal of Pharmacopuncture, the Journal of Acupuncture Research, the Korean Journal of Acupuncture, the Journal of Oriental Rehabilitation Medicine, the Journal of Korean Medicine, and the Journal of Korea CHUNA Manual Medicine for Spine and Nerves.

The search terms will consist of a combination of “pharmacopuncture” and “adverse events”. Synonyms of pharmacopuncture (herbal acupuncture, acupoint injection, aqua acupuncture, and aquapuncture) and adverse events (adverse reaction, side effects, risk, or safe) will also be searched. The search strategy will be modified appropriately according to each database (Table 2).

**Table 2:** Search strategy for the databases.

DBs	No.	Search terms
CENTRAL	#1	MeSH descriptor: [Acupuncture] explode all trees
	#2	(pharmacopuncture^∗^): ti,ab,kw OR (“herbal acupuncture”): ti,ab,kw OR (“aqua acupuncture”): ti,ab,kw OR (aquapuncture^∗^): ti,ab,kw OR (“acupoint injection”):ti,ab,kw (Word variations have been searched)
	#3	MeSH descriptor: [Risk] explode all trees
	#4	MeSH descriptor: [Safety] explode all trees
	#5	(“adverse events”) OR (“side effects”) OR (“adverse reaction”) OR (safe^∗^) OR (risk^∗^) (Word variations have been searched)
	#6	#1 OR #2
	#7	#3 OR #4 OR #5
	#8	#6 AND #7
PUBMED	#1	(pharmacopuncture^∗^ [Title/Abstract]) OR (aquapuncture^∗^ [Title/Abstract]) OR (“aqua acupuncture” [Title/Abstract]) OR (“herbal acupuncture” [Title/Abstract]) OR (“acupoint injection” [Title/Abstract])
	#2	(side effects [MeSH Terms]) OR (adverse effects [MeSH Terms])
	#3	“adverse events” OR “adverse reaction” OR “adverse effects” OR “side effects” OR safe^∗^ OR risk^∗^
	#4	#1 AND (#2 OR #3)
EMBASE	#1	“pharmacopuncture”/exp
	#2	pharmacopuncture^∗^: ti,ab,kw OR aquapuncture^∗^: ti,ab,kw OR ’aqua acupuncture’:ti,ab,kw OR ’herbal acupuncture’:ti,ab,kw OR “acupoint injection”: ti,ab,kw
	#3	“adverse event”/exp OR “side effect”/exp OR “safety”/exp OR “risk”/exp
	#4	“adverse events”: ti,ab,kw OR “adverse reaction”: ti,ab,kw OR “side effects”: ti,ab,kw OR safe^∗^:ti,ab,kw OR risk^∗^:ti,ab,kw
	#5	#1 OR #2
	#6	#3 OR #4
	#7	#5 AND #6
Korean DBs^∗^	#1	(in Korean) Yakchim (Acupuncture; pharmacopuncture) (ti,ab,kw, MESH)
	#2	(in Korean) Boojakyong (Side effects, abnormal; adverse events) (ti,ab,kw, MESH)
	#3	pharmacopuncture (e.g., acupoint injection, aquapuncture, aqua acupuncture, herbal acupuncture) (ti,ab,kw, MESH)
	#4	“adverse events” (e.g., side effect, risk, safe) (ti,ab,kw, MESH)
	#5	#1 AND #2
	#6	#3 AND #4
	#7	#5 OR#6
Google Scholar	intitle:pharmacopuncture AND (“adverse events” OR “side effect” OR risk OR safe)	

### 2.3 Data collection and analysis

#### 2.3.1 Study selection

**Figure 1:**
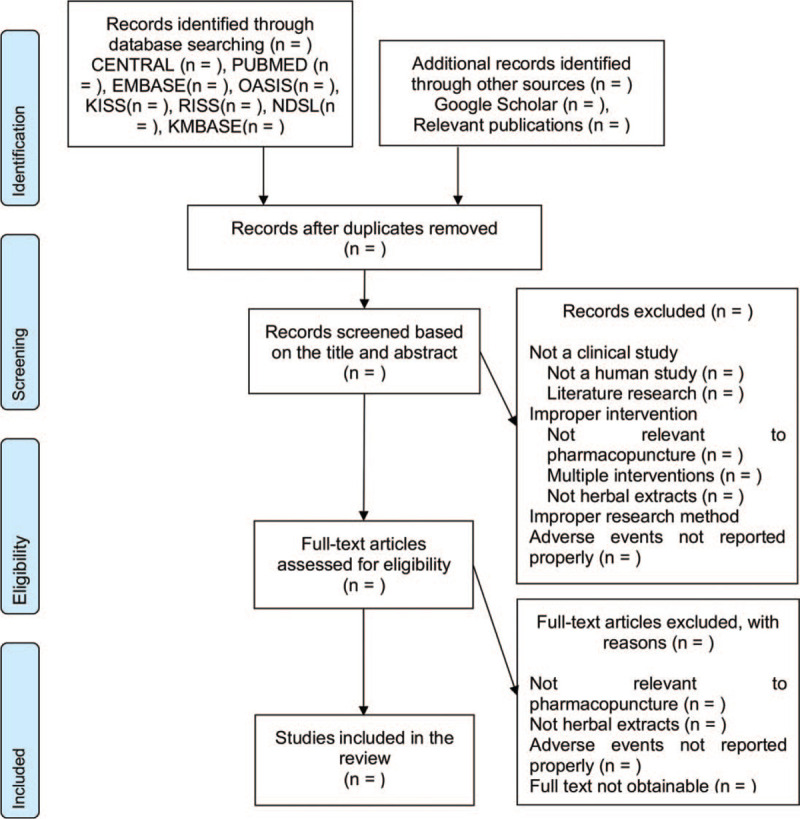
Flow chart of the study selection.

Two reviewers will independently examine the articles for inclusion based on the title and abstract. If there are disagreements regarding the selection, and it cannot be resolved through a discussion, an arbitrator will make the final decision (Fig. 1).

#### 2.3.2 Data extraction and management

Two reviewers will read the full text of the articles and independently extract data according to standard data extraction forms. All disagreements will be resolved by an agreement between the 2 reviewers, and the final data will be verified by another reviewer. If the articles included contain insufficient or ambiguous information, we will contact the corresponding authors by email or phone for further details.

#### 2.3.3 Risk of bias assessment

Two reviewers will evaluate the risk of bias independently using the Cochrane Collaboration's “risk of bias” tool for RCTs and the “Risk of Bias for Non-randomized Studies” tool for CCTs.^[14,15]^ If there are any disagreements between the 2 reviewers, an arbitrator will make the final decision.

### 2.4 Statistical analyses

Studies will be assessed for heterogeneity by Higgins's *I*^2^ statistics (significance level = 0.1). If the *I*^2^ value is 50% or less, a meta-analysis will be performed using the fixed-effect model. If the *I*^2^ value is higher than 50%, a random-effect model will be used for data pooling. If meta-analysis is not appropriate, we will conduct a narrative analysis.

### 2.5 Publication bias analysis

For included RCTs, the risk of publication bias will be assessed and expressed in the form of a contour-enhanced funnel plot.^[16]^ If the drawn funnel plot shows a possibility of substantial bias, Egger test will be conducted to evaluate the existence of publication bias.^[17]^ If less than 10 RCTs are included, tests will not be conducted due to the limited power of tests.

## 3 Discussion

There are a few systematic reviews on the AEs of pharmacopuncture, but the majority have not been adequately evaluated using international standards; therefore, it makes becomes difficult to assess the safety of pharmacopuncture. One study examines all pharmacopuncture types but is limited in that the AEs were not the main subject. Only RCTs were included, and they were not adequately evaluated. Additionally, the number of RCTs with AEs was small (5 out of 29 RCTs).^[18]^ Another study only examines bee venom, which is a type of pharmacopuncture. Its usefulness is limited in that the risk of bias was not evaluated.^[7,19]^

We will systematically collect and evaluate the frequency and patterns of AEs of pharmacopuncture according to international standards. This review will provide more concrete evidence regarding the safety of pharmacopuncture by comprehensively investigating all types of clinical studies in Korea. This will be useful for traditional medical doctors and patients who use such treatments and interventions.

However, this study has some limitations. First, this review includes only the studies that used pharmacopuncture with herbal extracts to better differentiate between Chinese acupoint injections and Western medicine. Additionally, only studies conducted in Korea will be included. Nevertheless, this study should be able to improve knowledge on the safety of pharmacopuncture for traditional doctors working outside of Korea. Additionally, this review may help to improve the reporting of AEs in future pharmacopuncture research.

Although pharmacopuncture has been extensively used by Korean medical doctors, the risks associated with pharmacopuncture have not been rigorously evaluated. We have prepared a protocol to gather information on the AEs associated with pharmacopuncture. This systematic review will provide clinicians and future researchers with a comprehensive understanding about the safety of pharmacopuncture.

## Author contributions

**Conceptualization:** Jaeeun Park.

**Data curation:** Jaeeun Park.

**Formal analysis:** Jaeeun Park.

**Investigation:** Jaeeun Park.

**Methodology:** Jaeeun Park.

**Project administration:** Jaeeun Park.

**Resources:** Jaeeun Park.

**Software:** Jaeeun Park.

**Supervision:** Yong-Cheol Shin, Seong-Gyu Ko.

**Validation:** Sohyeon Kang.

**Visualization:** Sohyeon Kang.

**Writing – original draft:** Jaeeun Park.

**Writing – review & editing:** Bo-Hyoung Jang.
